# Biomechanical analysis of spinal cord injury during scoliosis correction surgery

**DOI:** 10.3389/fbioe.2024.1399691

**Published:** 2024-07-02

**Authors:** Haimei Wang, Chunyu Zhang, Yongqiang Wang, Yan Zeng, Songhao Chen, Xingyu Su, Weishi Li, Miao Yu, Duanduan Chen

**Affiliations:** ^1^ School of Medical Technology, Beijing Institute of Technology, Beijing, China; ^2^ Department of Orthopaedics, Peking University Third Hospital, Beijing, China; ^3^ College of Engineering, Peking University, Beijing, China

**Keywords:** scoliosis, surgery, spinal cord, poor prognosis, finite element model

## Abstract

**Introduction:** Surgical correction is a common treatment for severe scoliosis. Due to the significant spinal deformation that occurs with this condition, spinal cord injuries during corrective surgery can occur, sometimes leading to paralysis.

**Methods:** Such events are associated with biomechanical changes in the spinal cord during surgery, however, their underlying mechanisms are not well understood. Six patient-specific cases of scoliosis either with or without spinal complications were examined. Finite element analyses (FEA) were performed to assess the dynamic changes and stress distribution of spinal cords after surgical correction. The FEA method is a numerical technique that simplifies problem solving by replacing complex problem solving with simplified numerical computations.

**Results:** In four patients with poor prognosis, there was a concentration of stress in the spinal cord. The predicted spinal cord injury areas in this study were consistent with the clinical manifestations of the patients. In two patients with good prognosis, the stress distribution in the spinal cord models was uniform, and they showed no abnormal clinical manifestations postoperatively.

**Discussion:** This study identified a potential biomechanical mechanism of spinal cord injury caused by surgical correction of scoliosis. Numerical prediction of postoperative spinal cord stress distribution might improve surgical planning and avoid complications.

## 1 Introduction

The normal spine curves inwardly in the cervical region, outwardly in the thoracic region and inwardly again in the lumbar region. Scoliosis, a complex deformity of the trunk, typically manifests as a three-dimensional malformation of the spine (Cheng et al., 2015). This malformation is reflected in deviations from the median line on the coronal plane, and abnormal kyphotic curve on the sagittal plane (Wang et al., 2017; YE et al., 2017; Liu et al., 2019). A patient with severe scoliosis may experience heart and lung problems (Janicki and Alman, 2007) and in severe cases, restricted movement or complete paralysis of the lower limbs may occur (Clark and Bracci, 2019). Patients with severe scoliosis often require surgical implantation of pedicle screws, hooks, and rods to attempt to restore the spine to its natural position through force and displacement (Maruyama and Takeshita, 2008; Mundis and Akbarnia, 2010). However, such surgeries are invasive, difficult and can cause permanent spinal cord damage that may result in reduced mobility of the extremities or paraplegia (Weiss and Goodall, 2008; Halsey et al., 2020). A 2-year follow-up study by Bartley et al., on 2,220 patients undergoing spinal correction surgery reported a complication rate of 4.1%, with neurological injuries accounting for 0.5%, and device-related complications for 0.4% (Bartley et al., 2017).

During scoliosis correction surgery, evoked potentials are used to measure electrical activity within the spinal cord (Sakaki et al., 2012). Somatosensory evoked potential (SSEP) and motor evoked potentials (MEPs) are frequently utilized to monitor spinal cord health during surgery (MacDonald, 2006; Cruccu et al., 2008). SSEP measures bioelectric responses within specific pathways of the body’s afferent sensory system upon stimulation (Eisen, 1982). A decreased SSEP signal can indicate spinal cord damage. MEPs are electrical signals recorded from the muscles upon stimulation of the motor cortex. However, descending efferent spinal cord motor activity cannot be directly measured with MEPs(Frost, 2015) and it may take time for spinal cord injury to manifest after scoliosis correction surgery (Diaz and Lockhart, 1987). Nerve trauma can lead to prolonged SEP latency, and if the latency exceeds 10% or the amplitude of the electrical potential decreases by more than 50%, it is necessary to interrupt the surgery to assess the risk factors for spinal cord injury (Hilgart et al., 2023).

The recent development and application of numerical computing technology has a significant impact on the prediction of surgical operations. The finite element (FE) simulation permits the assessment of biomechanical changes that occur during certain medical procedures as well as the physiological responses that occur as a result (Kaku et al., 2004). The stress in the spinal cord during orthopedic surgery cannot be accurately predicted, so there may be a risk of spinal cord injury. Use of FE methods during surgery (Greaves et al., 2008; Henao et al., 2016) can help minimize the drawbacks of inadequate experimental samples, low repeatability, and high cost and permits acquisition of precise stress distribution data within the spinal cord. Kim et al. (Kim et al., 2009) investigated the effects of scoliosis on nerve roots using an FE model of computed tomography (CT) images of a healthy subject, in which they simulated scoliosis by moving the spine; however, their model was based on a healthy people, they did not take into account the specificities of various patients. FE mechanical modeling in the assessment of spinal cord damage were also employed by Shervin et al. (Jannesar et al., 2021). Despite numerous studies, the biomechanics of spinal cord injury during scoliosis correction surgery remain uncertain. Further, there is a need for a clinical tool to assess spinal cord biomechanics during corrective scoliosis surgery, predict the possible location of spinal cord damage, and assist surgeons in making a plan to minimize risk of injury.

Here, we created three-dimensional FE models specifically matched to the spinal cords of study patients in order to examine spinal cord stress distribution during scoliosis correction surgery. These findings may help clinicians better predict the spinal segments where neurological complications could occur during surgery.

## 2 Materials and methods

### 2.1 Patients

This study included six scoliosis patients undergoing corrective spinal surgery for scoliosis. All participants provided signed informed permission forms and the study was approved by the Institutional Review Board of the Peking University Third Hospital (S2021442). Patients received CT and magnetic resonance imaging (MRI) scans before and after surgery.

Pre-surgery CT and MRI scans were performed 2 days before scoliosis correction surgery and post-surgery scans were performed within 4 days of the procedure. Post-surgery scans showed that no screws invaded the spinal canal to cause further spinal cord compression. MRIs showed no hematoma or other abnormalities in the spinal cord. Among patients a to d, those with poor prognoses include patient a, who experienced complete loss of light touch and acupuncture sensation below the T8 level, leading to clinical diagnosis of paraplegia at the T8-T9 level. Patient b had decreased or absent acupuncture sensation at and below bilateral T6 levels, patient c exhibited weakened lower limb mobility, and patient d suffered thoracic spinal cord damage resulting in incomplete loss of lower limb function during surgery. Conversely, patients e and f had a positive prognosis.

### 2.2 Creating the spinal cord models of scoliosis

**FIGURE 1:**
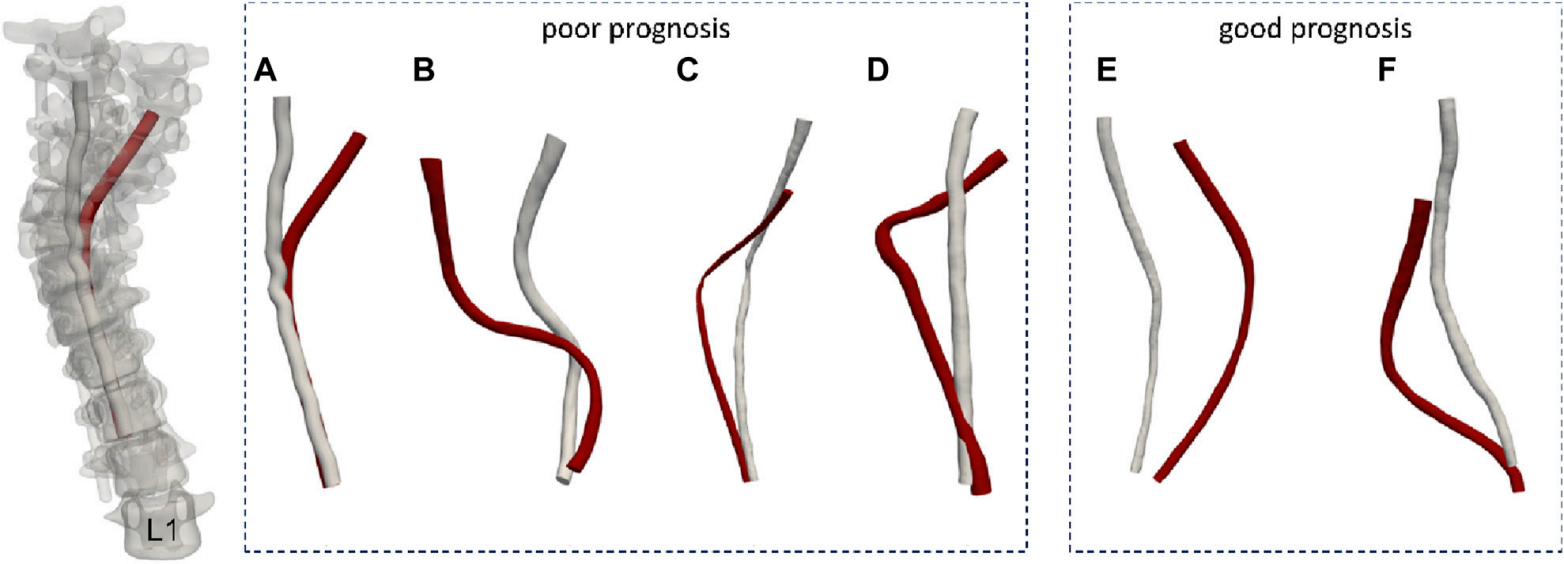
The spinal cord models of the patients. (**A-D** patients with poor prognosis, **E, F** patients with good prognosis. The red model represents the preoperative spinal cord morphology, while the white model illustrates the postoperative spinal cord morphology.)

Spinal cords were modeled using cross-sections of patient MRI images (GE Medical Systems, USA). Scan parameters are as follows: voltage 120 kV, kilovolt peak 120kvp, and slice thickness 5 mm. Three-dimensional patient-specific models were produced by medical image processing software (Mimics 19.0; Materials, Leuven). Patients’ DICOM CT images were imported into Mimics, and a spinal model was extracted through application of bone threshold segmentation, regional growth, and manual editing. After importing DICOM images from MRI scans into Mimics, 3D spinal cord models were created utilizing manual editing and threshold processing. The models were rough compared to actual spinal cords, which were relatively smooth, so reverse engineering software was utilized to optimize construction using denoising and smoothing techniques in Geomagic Wrap (Geomagic Wrap 2021, 3D Systems, United States). Smoothed-out representations of the spinal cord models are shown in Figure 1.

### 2.3 Mesh generation and material property assignment

Spinal cord models were discretized using tetrahedral grids. Patient a has a spinal cord FE model length of 188 mm, corresponding to the region between the second thoracic vertebra and the first lumbar vertebra. The discrete model has 1,499 nodes. Patient b’s model length is 241 mm, covering the segment from the first thoracic vertebra to the first lumbar vertebra, with 2,922 nodes. Patient c’s model length is 287 mm, covering from the first to the 12th thoracic vertebra, with 2,312 nodes. Patient d corresponds to a 168 mm spinal cord model covering the first to the 11th thoracic vertebra, with 1,680 nodes. Patient e’s model length is 241 mm, covering the segment from the first thoracic vertebra to the first lumbar vertebra, with 2,566 nodes. Patient f corresponds to a 245 mm spinal cord model covering the first to the 11th thoracic vertebra, with 3,529 nodes. All models were regarded as a homogeneous isotropic substance with Young’s modulus of 0.26 MPa and a Poisson’s ratio of 0.49 (Bassi et al., 2009; Xue et al., 2021; Sim et al., 2022).

### 2.4 Boundary conditions

Due to the different imaging principles and application ranges of CT and MRI, MRI can effectively show soft tissues such as the spinal cord’s morphology, while CT provides better imaging of bones. The CT and MRI data of each patient in this study were acquired at different times with different equipment performance and technical parameters. The spinal cord and spine models of the same patient before and after surgery are in different coordinate systems. To ensure the accuracy of the simulation, registration is required. The vertebrae have high hardness, and spinal correction surgery causes minimal changes in their morphology. Therefore, in this study, the vertebrae are used as the registration standard to confirm the correct position of the spinal cord in the vertebral foramen.

For the accurate registration of the spinal cord, this study outlined the spinal model’s contour in the patient’s MRI images and marks it along with the spinal cord model. Six files (preoperative spinal cord model, postoperative spinal cord model, preoperative spine model, postoperative spine model, preoperative spine contour, postoperative spine contour) were imported into 3-matic. Using the postoperative spinal cord coordinate system as the reference coordinate system, registration was performed with the first lumbar vertebra (L1) as the reference vertebra. The postoperative spinal cord model and spine contour were bound, and the postoperative spine model was moved so that the L1 of the postoperative spine model coincides with the L1 of the spine contour. Based on the anatomical features of the spinal cord and spine, segmentation checks were performed to confirm that the preoperative spinal cord model was in the correct position in the corresponding vertebral foramen of the spine model. The postoperative spinal cord model was then moved to the corresponding position in the vertebral foramen of the spine model using the same method. To achieve an accurate comparison between preoperative and postoperative imaging data, the preoperative spinal cord model and preoperative spine model were co-registered, synchronously moving them so that the L1 of the preoperative spine model coincides with the L1 of the postoperative spine model, establishing the spatial relationship between the spinal cord models before and after surgery. The registered spinal cord model was saved as a new stl (registered) model for further step.

The preoperative and postoperative spinal cord models were loaded into Mimics software, and the centerline generation tool was used to analyze the images, extracting the central axis of the spinal cord model. To more accurately quantify the morphological response of the spinal cord in corrective surgery, an interpolation algorithm was used to optimize the central line by sampling the coordinates at 0.5 mm intervals along the central line, obtaining a series of uniformly distributed coordinate points. Based on each coordinate point on the central line of the spinal cord, this study analyzed and extracted valuable information in three-dimensional space for each point. Initially, the study located each central point and extracted three-dimensional cross-sections of the spinal cord in the direction perpendicular to the segment of the central line where the point was located. These cross-sections reflected the morphology of the spinal cord at different positions. The study further quantitatively analyzed the spinal cord cross-sections, extracting area information and normal vectors for each cross-section to ensure the accurate reproduction of the position and three-dimensional morphology of each spinal cord cross-section.

**FIGURE 2:**
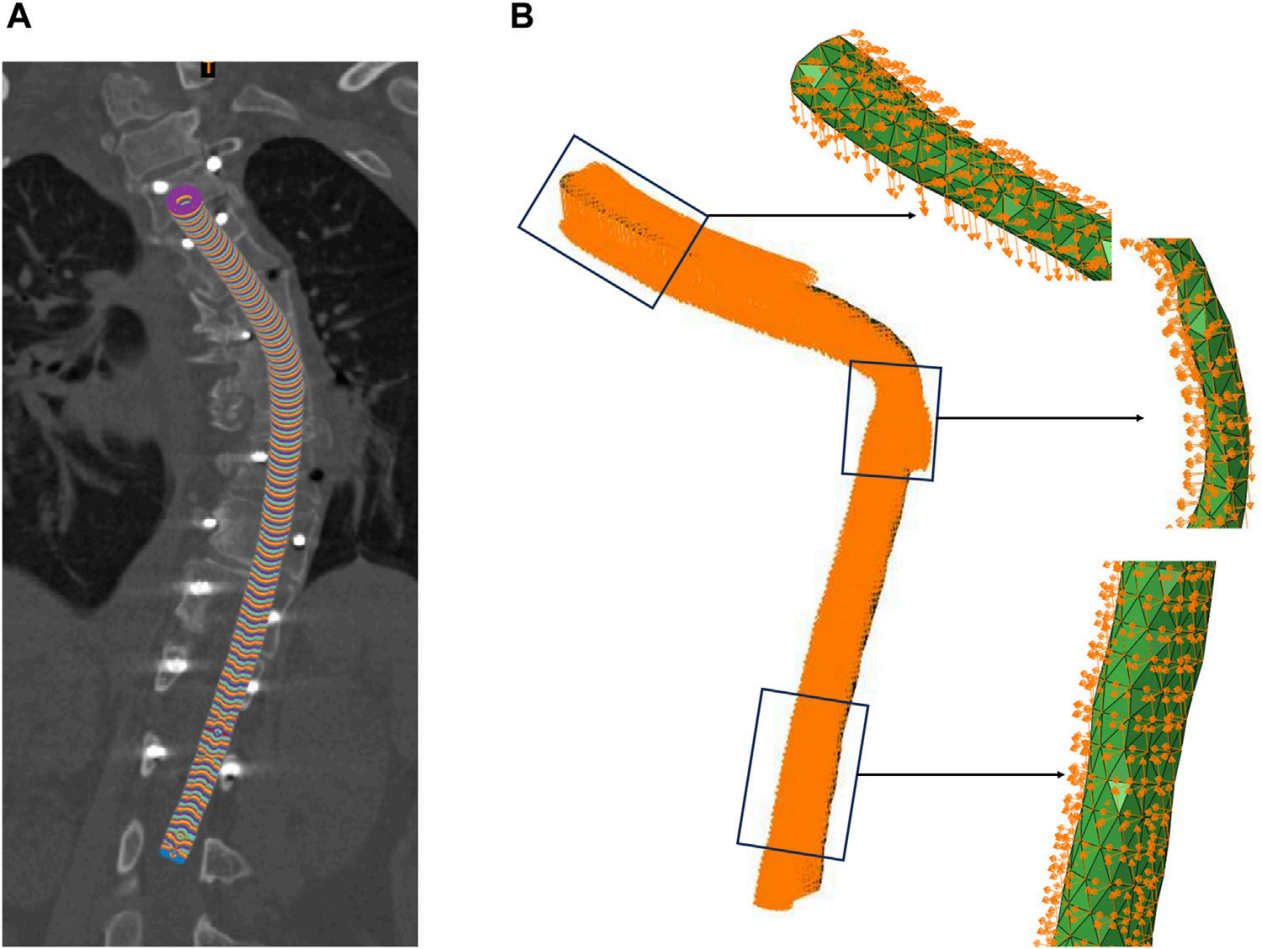
Boundary Conditions. **(A)** The preoperative spinal cord tube of patient, **(B)** Displacement of the spinal cord nodes.

Due to significant morphological differences in the spinal cord before and after surgery, accurately aligning the coordinate systems of the spinal cord prior to and following surgery constituted a core challenge of this study. A novel three-dimensional circular pipe modeling method, grounded in spinal cord morphology restoration, was proposed herein. For each spinal cord model, a virtual circular pipe model was constructed in three-dimensional space, leveraging the central line coordinates and normal vectors, thereby simulating the three-dimensional morphology of the spinal cord in space. The preoperative spinal cord pipe for patient e is depicted in the illustration provided. For each three-dimensional circular conduit mirroring the spinal cord, a set of data points was sampled along the circumference of each cross-sectional circle at 0.5-mm intervals in a predetermined direction (Figure 2A). These sampled data points collectively and precisely replicated the circular profile of the cross-section. In order to further acquire a sequence of coordinates within the conduit, a radial decremental calculation was executed at 0.5-mm intervals, progressively narrowing the circumference of the three-dimensional conduit to extract interior coordinate points, thus giving rise to a comprehensive set of digitized circular sequence coordinate data. At this juncture, the coordinate sequence of the preoperative spinal cord conduit model for every patient corresponded one-to-one with the postoperative spinal cord conduit model’s coordinate sequence in terms of physiological structure.

In this study, to procure boundary conditions, attaining meticulous matching and linkage between the coordinates of each node in the preoperative spinal cord finite element model (FEM) and the respective three-dimensional conduit coordinate sequence, previously stored and embodying the genuine anatomical locales of the spinal cord, was essential. An algorithm was devised to achieve this objective, harmonizing the preoperative spinal cord FEM coordinate sequence with the antecedently stored conduit coordinate sequence data via geometric mapping, mathematical interpolation, iterative optimizations, among other computations. This guaranteed that the node coordinate ensemble within the spinal cord FEM conformed to the corresponding surface or intrinsic data coordinates of the spinal cord conduit, instituting a one-to-one correlation and designating the earmarked spinal cord conduit coordinates.

Building upon this foundation, the aim was to systematically identify and correlate the pre-labeled preoperative spinal cord conduit coordinates with the postoperative spinal cord conduit coordinates. Through precise algorithmic matching, a comparative analysis was conducted between the two independent sets of coordinate data (the pre-labeled preoperative spinal cord conduit coordinates and the postoperative spinal cord conduit coordinates), extracting the postoperative spinal cord conduit coordinates corresponding to the pre-labeled preoperative spinal cord conduit coordinates, and then calculating the relative displacement of each node in the three coordinate axes. This algorithm successfully facilitated the continuous quantitative analysis of the patient’s spinal cord morphology from preoperative to postoperative states, providing the boundary conditions for dynamic FEA of the spinal cord in this study. The diagram illustrated the boundary conditions of the malformed region of patient e’s spinal cord, including the displacement in the x, y, and z directions for each node.

### 2.5 Finite element analysis

FEA is indeed a powerful numerical technique that simplifies problem solving by breaking down complex problems into manageable components. In the context of spinal cord research, FEA and the construction of three-dimensional FEMs play a crucial role in understanding the biomechanical changes that occur during the surgical process. Developing high-precision spinal cord models is essential for accurately capturing the stress and strain experienced by the spinal cord during surgery. In this study, the displacement changes of the spinal cord model before and after surgery are simulated to model spinal cord motion during scoliosis correction surgery. The Abaqus CAE FE solver (Abaqus CAE 2020; USA) is used to solve the mechanical components of the FEM. Based on the von Mises theory, the von Mises equivalent stress is selected as the analysis metric for computing results. In complex stress states, materials exhibit plasticity when the shape change at a point in the material exceeds the limit of the energy change compared to uniaxial yield. The preoperative spinal cord discrete model node displacements (Figure 2B) are input into the Abaqus CAE software’s load module, and the job is submitted for calculation to obtain the stress on the spinal cord during surgery. The results of the FE calculations are analyzed by selecting the maximum stress value and stress distribution within the region of interest.

### 2.6 Model validation

**FIGURE 3:**
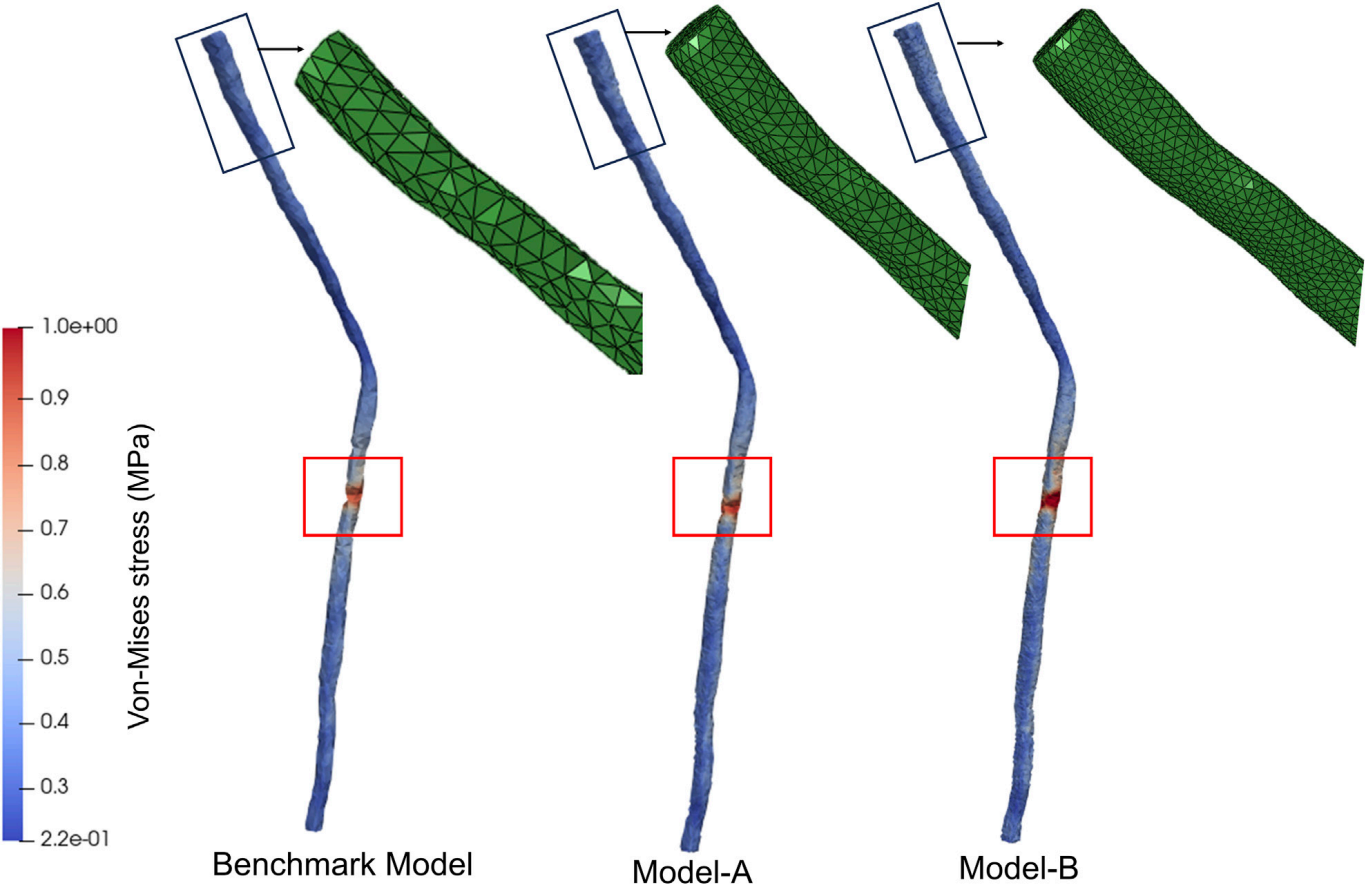
Model validation. Stress distribution in spinal cord models with different mesh sizes.

To confirm the insensitivity of the results to the spatial resolution of the mesh, a mesh independence analysis was conducted. Given that the boundary conditions of this study were based on the node displacements of the discrete model, model validation in this study was conducted based on the number of nodes. In addition to the basic discretization, a more refined mesh with 8,770 nodes (Model-A) and 17,812 nodes (Model-B) was studied, representing a refinement of 5 times and 10 times compared to the baseline model. To compare the results of these three models, the morphological changes of the spinal cord during spinal deformity correction surgery were used as boundary conditions to compare the stress distribution characteristics of the three models. The stress distribution features of Model-A and Model-B were found to be similar to those of the baseline model, with stress concentration occurring in the T8-T9 segment, as shown in Figure 3. The maximum difference in the maximum equivalent stress between the three FEMs was within 10%. Therefore, in our study, it was considered that discretizing the spinal cord into 2,312 nodes was sufficient.

## 3 Results

Since the spine and spinal cord segments do not correspond directly, vertebral segments were used by physicians to determine the location of spinal cord injury. As a result, relabeled spinal cord segments were used for stress-strain analysis in this study.

### 3.1 Displacement of the spinal cord

**FIGURE 4:**
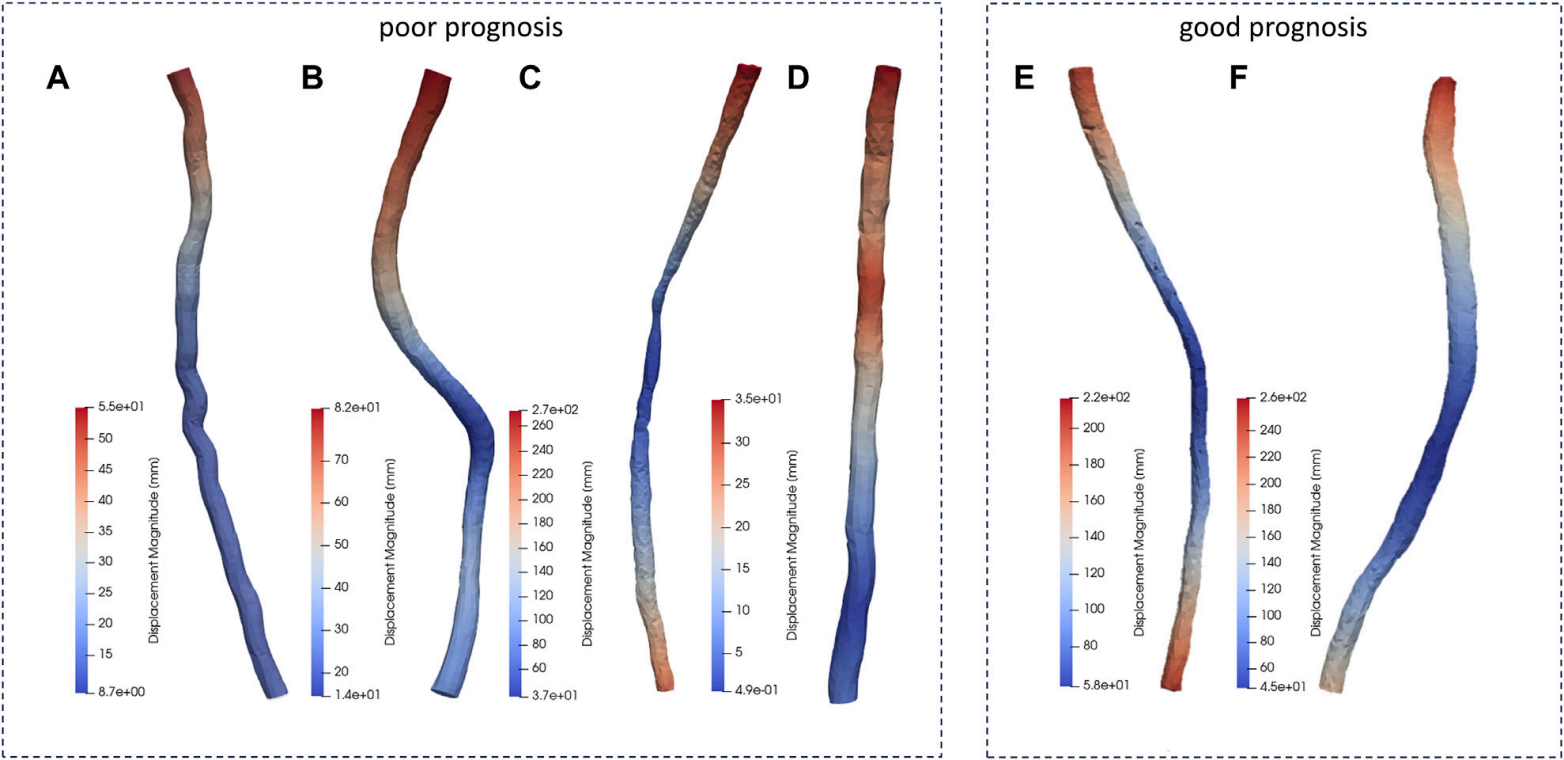
Displacement distribution of the patients. (**A-D** patients with poor prognosis, **E, F** patients with good prognosis.)

In this study, biomechanical changes of the spinal cord during surgery performed to repair deformities resulting from scoliosis were simulated by FE modeling. Changes in displacement of patients’ spinal cords during scoliosis corrections are shown in Figure 4. The maximum spinal cord displacement in the dynamic simulation was highly correlated with the maximum spinal cord displacement mirrored in medical imaging data (as per Table1, *p* < 0.05), validating this study’s capability to reproduce spinal cord morphological alterations during corrective interventions. The sites of maximum displacement for the spinal cord models pertaining to patients a through f were respectively identified at T2, T1, T1, T1, L1, and T1.

**TABLE 1:** Maximum spinal cord displacement (mm) in medical images and dynamic simulations.

Patients	A	B	C	D	E	F
medical image	54.78	82.47	271.75	34.23	210.45	264.09
dynamic simulation	55.14	82.12	272.44	35.04	210.41	264.67

### 3.2 Stress distribution of the spinal cord

Patient c exhibited a maximum stress value reaching up to 0.921 MPa, hinting that heightened stress levels could be a factor contributing to spinal cord injury. Patients a, with 0.271 MPa, b, with 0.275 MPa, and d, with 0.255 MPa, demonstrated significantly lower peaks of maximum stress in contrast to patients e, at 0.617 MPa, and f, at 0.622 MPa. This variance suggested that the biomechanical mechanisms driving spinal cord injury were not adequately captured by considering maximum stress parameters alone. Hence, this investigation proceeded to undertake a more thorough examination of the internal stress distribution characteristics within the spinal cords of the patients, seeking to explain the biomechanics of spinal cord injury based on these stress distribution features.

**FIGURE 5:**
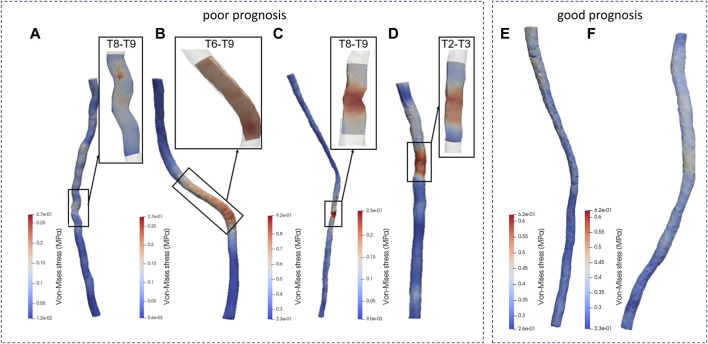
Von-Mise distribution of the patients. (**A-D** patients with poor prognosis, **E, F** patients with good prognosis.)

Patient a showed stress concentration in the spinal cord at the T8-T9 segments (Figure 5), consistent with the observed area of neurological dysfunction in clinical practice. CT and MRI screening confirmed appropriate placement of pedicle screws without direct spinal cord compression or structural abnormalities at the laminectomy site. There was a clinical diagnosis of paraplegia at the T8-T9 segments. This conclusion aligns with the biomechanical simulation analysis of the spinal cord dynamics conducted in this study.

In analyzing the morphological changes in the spinal cord model simulation of patient b during surgery, it was found that there was stress concentration in the spinal cord model at the T6-T9 segments (Figure 5), consistent with the area of nerve injury observed clinically. Clinical diagnosis confirmed incomplete thoracic spinal cord injury as a complication following spinal correction surgery, with the injury range matching the symptoms and aligning with the predicted injury area (T6-T9 segments) in the spinal cord model. During the spinal correction process, there were local nerve structure damage and resulting in loss of sensory and motor function in both lower limbs.

The biomechanical analysis results of patient c in this study showed significant stress concentration in the T8-T9 spinal cord segments (Figure 5), consistent with postoperative clinical manifestations. Clinical evaluation confirmed weakened postoperative mobility of both lower limbs, in line with the biomechanical analysis results of the spinal cord model in this study.

Analysis of the spinal cord mechanical model of patient d in this study revealed significant stress concentration in the spinal cord at the T2-T3 segments (Figure 5), matching the observed distribution area of neurological dysfunction in clinical practice. Clinical assessment diagnosed thoracic spinal cord injury and incomplete loss of lower limb function during surgery, consistent with the biomechanical analysis results of the spinal cord model in this study.

Similarly, this study conducted dynamic simulations of personalized spinal cord models for two patients (e and f) undergoing spinal corrective surgery, to simulate the morphological changes of the spinal cord during the procedure. The simulation results, as shown in the figure, indicated uniform distribution of internal stresses within each spinal cord model, without significant stress concentration areas. Each patient underwent varying degrees of bone resection and internal fixation with pedicle screw fixation in different regions during the surgery, while maintaining a balanced biomechanical environment of the spinal cord throughout the procedure. In postoperative clinical assessments, two patients showed good prognosis with normal sensory and motor functions in the upper and lower limbs, as well as good joint mobility, consistent with the results predicted by the spinal cord simulations in this study.

## 4 Discussion

Previous FE studies involving scoliosis typically investigated biomechanics of the vertebral body; however, spinal surgery close to the spinal cord carries the risk of permanent damage. One of the most worrisome albeit uncommon side effects of scoliosis correction surgery is paraplegia, a loss of movement and sensation in the legs and lower body. During surgery, the spinal cord injury damage can be observed using electrophysiological techniques like SSEP and MEP; however, there may be a delay in onset of symptoms and detection of injury (MacDonald, 2006; Cruccu et al., 2008). The FE simulation model simulates the stress and strain of the spinal cord during scoliosis correction surgery, which is beneficial to the clinical understanding of spinal cord injury during such procedures. In addition, there remains a need for clinical tools that can be used to evaluate biomechanical changes of the spinal cord during surgery, predict locations where spinal cord injury might occur during surgery, and assist surgeons in pre-operative planning to reduce the risk of potentially permanent damage. Additionally, the specificity of the patients was taken into account, every patient had his own FEM, which can assist surgeons in preoperative surgical planning for different patients. The feasibility of the methodology used here was verified by comparisons with patient prognosis given by clinician and our findings are expected to direct and assess scoliosis surgery in the future.

Biomechanical analysis was carried out on each spinal cord section for all patients to further determine the segment of the spinal cord injured during surgery. The six patients were divided into two groups (poor prognosis group: a to d; good prognosis group: e, f), and three-dimensional spinal cord models were established for each group, with dynamic boundaries extracted for FE calculations, followed by detailed analysis of the results. The study revealed a high correlation between the maximum displacement in the simulated spinal cord models and the maximum displacement reflected in medical images, successfully replicating the morphological changes of the spinal cord during surgery.

Patient a’s spinal cord model exhibited stress concentration in the T8-T9 segment, corresponding to the clinical diagnosis of complete loss of light touch and pinprick sensation from the T8 level downwards, resulting in paraplegia at the T8-T9 segment, consistent with the dynamic simulation results of this study. Patient b’s spinal cord model showed stress concentration in the T6-T9 segment, consistent with the clinical diagnosis of sensory loss segment (T6 segment). Patient c’s spinal cord model indicated stress concentration in the T8-T9 segment, corresponding to postoperative clinical manifestations of reduced lower limb movement, consistent with the dynamic simulation results. For patient d, the spinal cord mechanical model revealed stress concentration in the T2-T3 segment, and the clinical diagnosis by doctor’s post-surgery indicated thoracic spinal cord injury, in line with the simulation results of this study.Similarly, dynamic simulations of the spinal cord for the two patients in the good prognosis group showed uniform distribution of internal stresses without stress concentration areas. The spinal cord model predictions in this study were highly consistent with clinical diagnostic results, successfully reproducing the morphological changes and stress distribution characteristics of the patients’ spinal cords during spinal corrective procedures using the developed dynamic boundary calculation method.

Maximum stress has been strongly correlated with spinal cord injury (Jannesar et al., 2021), which may explain the poor prognosis of these patients. Patients A and B had good prognoses, which aligned with our observation of no stress concentration in our simulation results. A high-precision spinal cord model is very important to reflect the stress and strain conditions of the spinal cord during surgery. However, due to the influence of implants, there may be significant artifacts in postoperative MRI scans (Garg et al., 2005; Koch et al., 2010). The cases included in this paper excluded the influence of artifacts, and postoperative spinal cord models more consistent with clinical reality were obtained.

This study has some limitations. In particular, while our FE models can accurately predict the damage of spinal segments of Patient C (with paraplegia) and Patient D (with reduced lower limb mobility), their utility is limited. Although the results of the FE methods were consistent with surgical records, the material properties of the spinal cords were not specific to any one patient, which was from the cadaver experiments. Manuel et al. studied the development of spinal cord mapping in human bodies and their findings may help address this issue, they created a probabilistic map and anatomical template of the human cervical thoracic spinal cord (Taso et al., 2014). In addition, the complexity of spinal cord tissue was not fully taken into account in this study. For example, white matter and gray matter have different pressure thresholds and ideally should be distinguished (Maikos et al., 2008). Previous studies have shown that blood vessel density in gray matter of the spinal cord is larger, capillaries are thinner, and that gray matter is more sensitive to injury (Hassler, 1966; Turnbull et al., 1966; Halder et al., 2018). In addition, the meninges and cerebrospinal fluid, which support and protect the spinal cord, can impact the FE modeling results and should be considered (Fradet, 2013; Jannesar et al., 2016). However, simulations involving CSF will involve calculation of fluid and solid coupling, which may increase computation time.

During surgery, spine deformity was reduced by force exerted by the implant, leading to stretching of the spinal cord. Specifically, compression between the implants led to stretching movement of the most deformed vertebrae in the sagittal plane (Owen et al., 1988; Jarzem et al., 1992). According to clinical studies, vertebral stretch has a greater impact on nerves than other manipulations (Shapiro et al., 2001; Bagchi et al., 2002); however, the magnitude and steps of spinal force and displacement applied during scoliosis correction surgery were not recorded here and this aspect was not studied. Nonetheless, these findings aid our understanding of the stress and strain conditions of the spinal cord during scoliosis correction surgery, which is valuable for scoliosis correction surgery planning and minimization of spinal cord damage. However, The feasibility of the method used in this study was validated by comparing it with patient outcomes given by clinicians, and our findings are expected to guide and evaluate future scoliosis surgery.

## 5 Conclusion

Patient-specific spinal cord models were established to analyze biomechanical changes including stress and strain on the spinal cords during scoliosis correction surgery in order to aid our understanding of spinal cord injury, predict the location of damage during surgery, and assist surgeons in making surgical plans to avoid injury and improve patient outcomes.

## Data Availability

The original contributions presented in the study are included in the article/Supplementary material, further inquiries can be directed to the corresponding authors.
